# Noise exposure alters MMP9 and brevican expression in the rat primary auditory cortex

**DOI:** 10.1186/s12868-020-00567-3

**Published:** 2020-04-25

**Authors:** Sung-su Park, Da-hye Lee, So Min Lee, Chang Ho Lee, So Young Kim

**Affiliations:** Department of Otorhinolaryngology-Head and Neck Surgery, CHA University College of Medicine, 59, Yatap-ro, Bundang-gu, Seongnam, Gyeonggi-do 13496 Korea

**Keywords:** Hearing Loss, Noise-induced, Brevican, Auditory cortex, Matrix metalloproteinase 9

## Abstract

**Background:**

This study aimed to investigate the changes in molecules related to perineuronal nets (PNNs) and synaptic transporters in the primary auditory cortices of rats with noise-induced hearing loss. Female Sprague–Dawley rats at postnatal day 7 were divided into the noise and control groups. Four hours of 115 dB SPL white noise was delivered for 10 days to the noise group. Thirty days after noise exposure, the primary auditory cortex and the inferior colliculus were harvested. The expression levels of vesicular glutamatergic transporter (VGLUT)1, VGLUT2, vesicular GABA transporter (VGAT), glutamate decarboxylase (GAD)67, brevican, aggrecan, MMP9, and MMP14 were evaluated using real-time reverse transcription polymerase chain reaction or western blot. An immunofluorescence assay was conducted to assess parvalbumin (PV), *Wisteria floribunda* agglutinin (WFA), and brevican. The immune-positive cells were counted in the primary auditory cortex.

**Results:**

The expression level of VGLUT1 in the primary auditory cortex was decreased in the noise group. The expression level of VGLUT2 in the inferior colliculus was elevated in the noise group. The expression levels of brevican and PV + WFA in the primary auditory cortex were decreased in the noise group. The expression level of MMP9 in the primary auditory cortex was increased in the noise group.

**Conclusion:**

Noise-induced hearing loss during the precritical period impacted PNN expression in the primary auditory cortex. Increased MMP9 expression may have contributed to the decrease in brevican expression. These changes were accompanied by the attenuation of glutamatergic synaptic transporters.

## Background

The deprivation of sensory stimulation, such as vision, leads to the decreased expression of perineuronal nets (PNNs) in the cortical area of the brain [1]. PNNs mainly surround parvalbumin (PV)-positive GABAergic interneurons [2]. By encasing PV-positive inhibitory interneurons, PNNs act as a barrier against oxidative injury and enhance the excitability of inhibitory neural transmission through interneurons [3]. Thus, the attenuation of PNNs may result in reduced inhibitory neural transmission and cortical disinhibition [4]. Similar to the visual system, auditory deprivation following noise exposure was shown to increase the excitatory neural transmission in the primary auditory cortex, which was presumed to result in cortical disinhibition and tinnitus [5]. Thus, it is speculated that the increased cortical excitability in a noise-induced hearing loss model could be related to the changes in PNNs.

Previous studies reported changes in PNNs in the primary auditory cortex following auditory deprivation [6, 7]. Noise-induced hearing loss in postnatal 4-week-old mice reduced PNNs, and this change was initiated as early as 1 day after noise exposure [6]. Although the density of PV-positive cells was not changed, the attenuation of PNN density lasted at least 30 days following noise exposure [6]. In the primary auditory cortex, PNNs deteriorate with age [8]. The development of PNNs is completed at approximately postnatal weeks 3–5, which coincides with the end of the critical period [1]. Thus, hearing impairment before the critical period might be accompanied by greater neuroplastic changes than that after the critical period. For instance, conductive hearing loss in the neonatal rat disrupted the development of PNNs in the superior olivary complex [9].

The present study predicted that noise-induced hearing loss would attenuate PNN expression in the primary auditory cortex. The decrease in PNNs may be associated with cortical neural transmission after noise-induced hearing loss. These neuroplastic changes in noise-induced hearing loss were anticipated to be greater before the critical period than after the critical period because previous studies described the development of PNNs following sensory experiences during the critical period, and the PNN expression levels were stable after the critical period [1, 8, 10]. In addition, prior studies demonstrated dynamic changes in PNNs during the first four postnatal weeks according to auditory deprivation [9]. Thus, rats with precritical period noise-induced hearing loss were investigated for changes in excitatory and inhibitory vesicular transporter systems in this study. In addition, the expression levels of PNNs and their degradative enzymes, matrix metalloproteinases (MMPs), were evaluated in the primary auditory cortex after noise exposure.

## Materials and methods

### Animals

The Institutional Animal Care and Use Committee of CHA University Medical School (IACUC170162) approved this study. All animal experiments were conducted in compliance with the guidelines and regulations of the Institutional Animal Care and Use Committee of CHA University Medical School.

Postnatal 7-day-old female Sprague–Dawley rats purchased from OrientBio were used [11]. Two rats were housed per standard cage. A 12-h light/dark cycle, temperature of 22–25 °C, and humidity of 50–60% were maintained. Water and food were freely accessible at all times.

### Noise-induced hearing loss paradigm

**Fig. 1:**
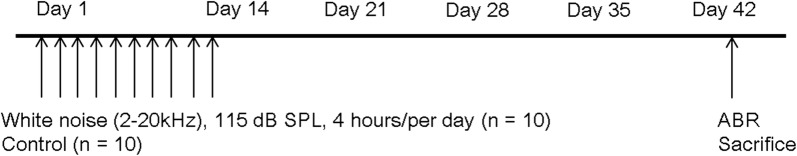
The design of the animal experiments. The noise group was exposed to white noise for 10 days. The brain was harvested 30 days after noise exposure

White noise (2–20 kHz, 115 dB SPL) was delivered to the white noise group for 4 h per day at 5 days per week for 2 weeks (n = 10) (Fig. 1). We used > 100 dB SPL noise to induce permanent threshold shift and exclude temporary threshold shift [12]. Noise was exposed throughout a sound chamber via free-field electrostatic speaker (Tucker-Davis Technologies, Alachua, FL, USA), which was located on top of the chamber. Rats were kept awake during noise exposure. Animals in the control group were raised under standard conditions and subjected to a background noise of approximately 40–60 dB SPL (n = 10). All rats were weaned at postnatal day 21.

### Auditory brainstem responses

Four weeks after white noise exposure, auditory brainstem responses (ABRs) were measured in all rats (SmartEP; Intelligent Hearing System, Miami, FL, USA). The needle electrodes were inserted into the vertex and behind the ipsilateral pinna. Plastic earphones were plugged into the right external auditory canals. The EC1 electrostatic speaker delivered 4, 8, 16, and 32 kHz of tone-burst stimuli (duration, 1562 µs; envelope, Blackman; stimulation rate, 21.1/s). The amplified evoked responses with 1024 sweeps were averaged. From 90 dB SPL, the tone-burst stimuli were decreased at 10 dB SPL intervals. The lowest sound intensity that evoked wave III (approximately 2–4 ms) was defined as the auditory threshold [13].

At 3–4 h after ABR recordings, the rats were sacrificed by carbon dioxide inhalation with flow rate of 5/6 L/min in 25.40 cm * 48.26 cm * 22.86 cm size cage. The carbon dioxide chamber was used according to previous guidelines for the euthanasia of rodents using carbon dioxide [14]. The primary auditory cortex and inferior colliculus were micropunched and rapidly frozen at − 20 °C (n = 5 for each group). These fresh frozen tissues were used for quantitative reverse transcription polymerase chain reaction (RT-PCR) (n = 5 for each group) and western blot (n = 5 for each group). For the immunofluorescence study, cardiac perfusion with phosphate-buffered saline was done under anesthesia using intraperitoneal injection of zoletil (40 mg/kg) and xylazine (10 mg/kg). Then, whole brain tissue was obtained and fixed in 4% paraformaldehyde (pH 7.4) (n = 5 for each group).

### Quantitative real-time reverse transcription polymerase chain reaction

The mRNA expression levels in the primary auditory cortex and inferior colliculus were analyzed using quantitative RT-PCR. TRI Reagent^®^ (Sigma–Aldrich, St. Louis, MO, USA) was used to extract total RNA from each brain tissue sample. Forward and reverse oligonucleotides were used for reverse transcription (Table 1), and mRNA expression levels were calculated. The target gene mRNA expression levels were expressed as a percentage of the glyceraldehyde 3-phosphate dehydrogenase mRNA expression levels.

**Table 1:** Oligonucleotide primer sequences for quantitative reverse transcriptase polymerase chain reaction

Gene	Primer sequence (forward)	Primer sequence (reverse)	Annealing temperature (°C)	Product size (bp)	RefSeq number
VGLUT1	5′-TTTCTACCTGCTCCTCATCTCC-3′	5′-ACACTTCTCCTCGCTCATCT-3′	60	574	NM_053859.2
VGLUT2	5′-CATGGTCAACAACAGCACCATC-3′	5′-CTCCCCGATGCTCTCTTCTATG-3′	60	574	NM_053427.1
VGAT	5′-GAAGAATCTCAAGGCCGTGTCCAA-3′	5′-CACGTAGATGGCCATGAGCAGCGT-3′	60	580	NM_031782.1
GAPDH	5′-GCGAGATCCCGCTAACATCA-3′	5′-CTCGTGGTTCACACCCATCA-3′	60	178	NM_017008.4

### Western blot analysis

The protein expression levels were analyzed by western blot. A radioimmunoprecipitation assay buffer (Cell Signaling Technology, Danvers, MA, USA) was used to lyse the brain tissue. The protein concentration was measured using a Bio-Rad Protein Assay Kit. The proteins were separated by 8% sodium dodecyl sulfate–polyacrylamide gel electrophoresis, transferred to polyvinylidene difluoride membranes (Merck Millipore, Burlington, MA, USA) and soaked in blocking buffer (5% nonfat dry milk in Tris-buffered saline containing Tween-20 [TBS-T]) for 1 h. Then, primary antibodies against VGLUT1 (#48–2400, rabbit polyclonal, Invitrogen), VGLUT2 (#ab79157, mouse polyclonal, Abcam, UK), GAD67 (#ab26116, rabbit monoclonal, Abcam, UK), aggrecan (#ab3773, mouse monoclonal, Abcam, UK), brevican (#ab111719, rabbit polyclonal, Abcam, UK), MMP9 (rabbit monoclonal, Abcam, UK), MMP14 (rabbit monoclonal, Abcam), and β-actin (D6A8, rabbit monoclonal; Cell Signaling Technology) were applied. Horseradish peroxidase (HRP)-conjugated secondary antibodies (#ab97023, goat anti-mouse IgG H&L, Abcam; #7074S, anti-rabbit IgG, Cell Signaling Technology) were used to detect the immunoreactive proteins, and the samples were visualized using an enhanced chemiluminescence kit (Bio-Rad). Protein expression levels were calculated using ImageJ gel analysis software (National Institutes of Health, Bethesda, MD, USA).

### Immunofluorescence

The regional expression of target proteins was examined using immunofluorescence staining. A paraffin block was made from dehydrated brain tissue and then cut into 10-µm-thick sections using a rotary microtome. Next, the tissue sections were mounted on glass slides, deparaffinized for 10 min in xylene, serially washed with ethanol (100%, 75%, and 50%) and washed with PBS 3 times for 5 min. The tissue sections were incubated in 10% donkey blocking serum (Vector Labs, Burlingame, CA, USA) for 1 h and then with the primary antibodies (rabbit anti-parvalbumin [PV27] and rabbit anti-brevican [#ab111719, Abcam, UK]) overnight at 4 °C. After three 10-min PBS washes, the slices were incubated with secondary antibodies (anti-rabbit Alexa 488 and 594) for 2 h. For PV + *Wisteria floribunda* agglutinin (WFA) immunostaining, the slices were incubated with fluorescein-labeled WFA (1:500; Vector Laboratories) in 2% normal goat serum (ab7481) overnight at 4 °C and then washed in PBS. The samples were incubated with 4′,6-diamidino-2-phenylindole (DAPI) (Sigma–Aldrich, St. Louis, MO, USA) for 5 min. After three 10-min washes with PBS, the slices were mounted on slides and covered.

The primary auditory cortex was localized according to Paxinos and Watson coordinates (A/P = − 2.7 ~ 5.8 mm, M/L = ± 6.4 ~ 8.7 mm) [15], and the densities of PV + WFA- and brevican-positive cells in cortical layers III-V were evaluated (5 rats per group, 2 slides per rats). A TCS SP5II confocal microscope (Leica, Wetzlar, Germany) was used. The numbers of PV + WFA- and brevican-positive cells per 350 × 250 mm^2^ were calculated. Thus, 10 images per group were analyzed. The cell counts were repeated by three researchers who were blinded to the study groups.

### Statistical analysis

The differences in gene expression and the densities and intensities of PV + WFA- and brevican-positive cells between the noise and control groups were analyzed by the Mann–Whitney U test using SPSS 21.0 (IBM Corp., Armonk, NY, USA). The differences in ABR thresholds between the noise and control groups were analyzed by T-test. The Statistical significance was considered as P < 0.05.

## Results

### Auditory threshold changes following noise exposure

**Fig. 2:**
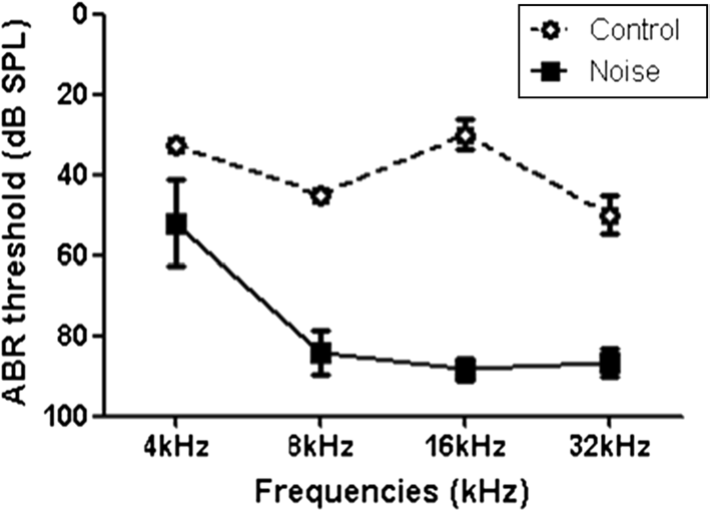
The auditory brainstem response (ABR) thresholds of the noise and control groups at 30 days after noise exposure. The noise group showed increased ABR thresholds at 4, 8, 16, and 32 kHz

The ABR thresholds were elevated in the noise group compared to the control group at 30 days after noise exposure (Fig. 2). The average ABR thresholds in the control group were 32.5, 45.00, 30.00, and 50.00 dB SPL at 4, 8, 16, and 32 kHz, respectively. The average ABR thresholds in the noise group were 52.08, 84.17, 88.33, and 86.67 dB SPL at 4, 8, 16, and 32 kHz, respectively (P < 0.001, degrees of freedom [d.f.] = 38, T-test for 4, 8, 16, and 32 kHz).

### Changes in the expression levels of vesicular synaptic transporters

**Fig. 3:**
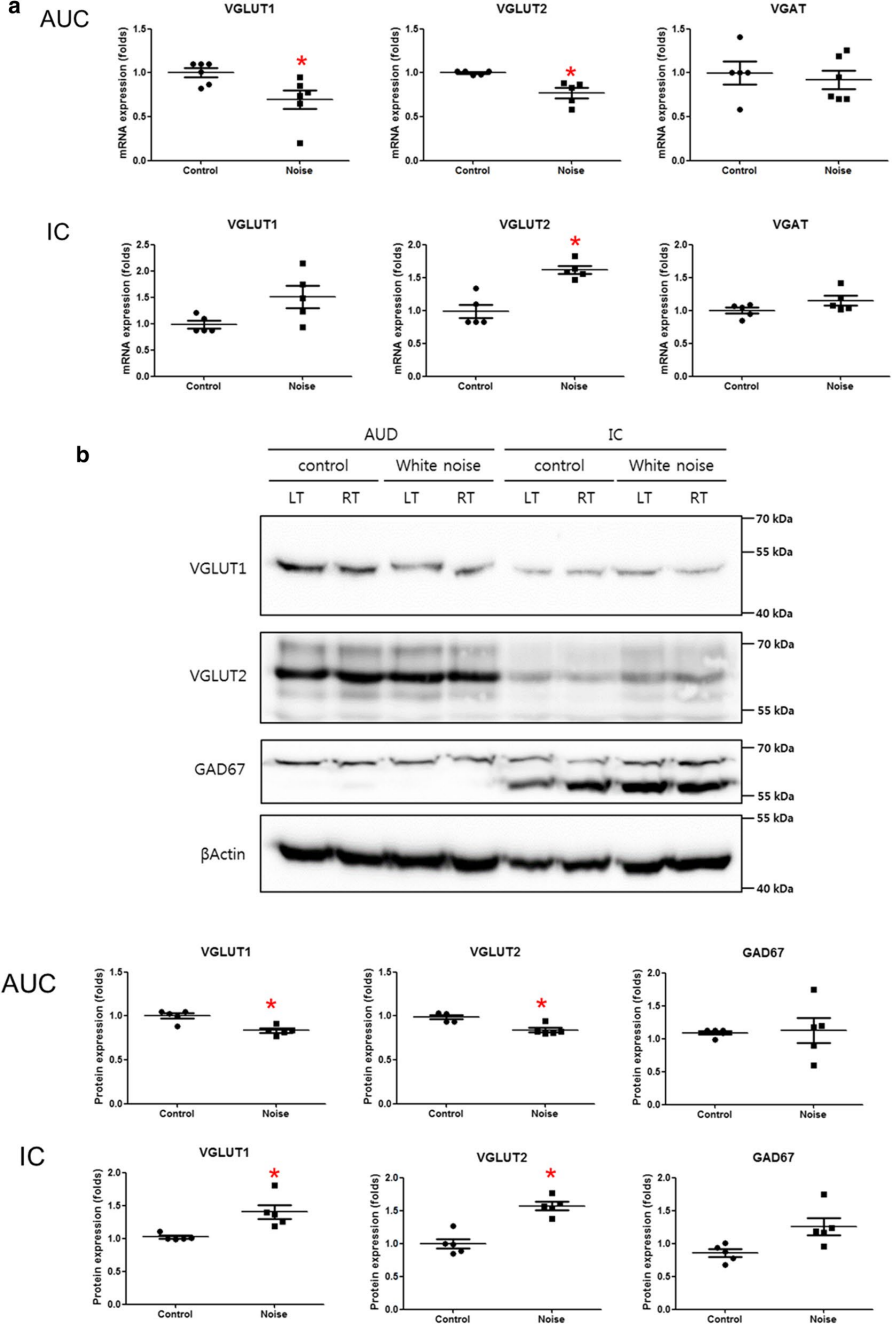
The expression of vesicular neural transporters in the primary auditory cortex and the inferior colliculus. **a** The mRNA expression levels demonstrated a decrease in VGLUT1 in the primary auditory cortex and an increase in VGLUT2 in the inferior colliculus in the noise group compared with the control group. **b** The protein expression levels showed a decrease in VGLUT1 in the primary auditory cortex in the noise group compared with the control group. (*LT* left, *RT* right, *AUD* auditory cortex, *IC* inferior colliculus) (*P < 0.05)

The mRNA expression level of VGLUT1 was decreased in the primary auditory cortex of the noise group (1.00 vs. 0.69 for control vs. noise groups, P = 0.02, d.f. = 8, Mann–Whitney U test) (Fig. 3). The protein expression level of VGLUT1 was also reduced in the primary auditory cortex of the noise group (1.00 vs. 0.83 for control vs. noise groups, P = 0.02, d.f. = 8, Mann–Whitney U test). The inferior colliculus demonstrated increased VGLUT1 mRNA expression in the noise group, but the difference was not statistically significant. The protein expression level of VGLUT1 in the inferior colliculus was not increased in the noise group. On the other hand, the mRNA expression level of VGLUT2 in the inferior colliculus was increased in the noise group (1.00 vs. 1.61 for control vs. noise groups, P = 0.01, d.f. = 8, Mann–Whitney U test). The protein expression level of VGLUT2 in the inferior colliculus was also increased in the noise group (0.99 vs. 1.57 for control vs. noise groups, P = 0.03, d.f. = 8, Mann–Whitney U test). The primary auditory cortex showed decreased VGLUT2 mRNA and protein expression levels (P = 0.01 for mRNA levels, P = 0.04 for protein levels, d.f. = 8, Mann–Whitney U test). The expression levels of VGAT and GAD67 did not differ between the noise and control groups in either the primary auditory cortex or the inferior colliculus.

### Changes in the expression levels of brevican and perineuronal nets

**Fig. 4:**
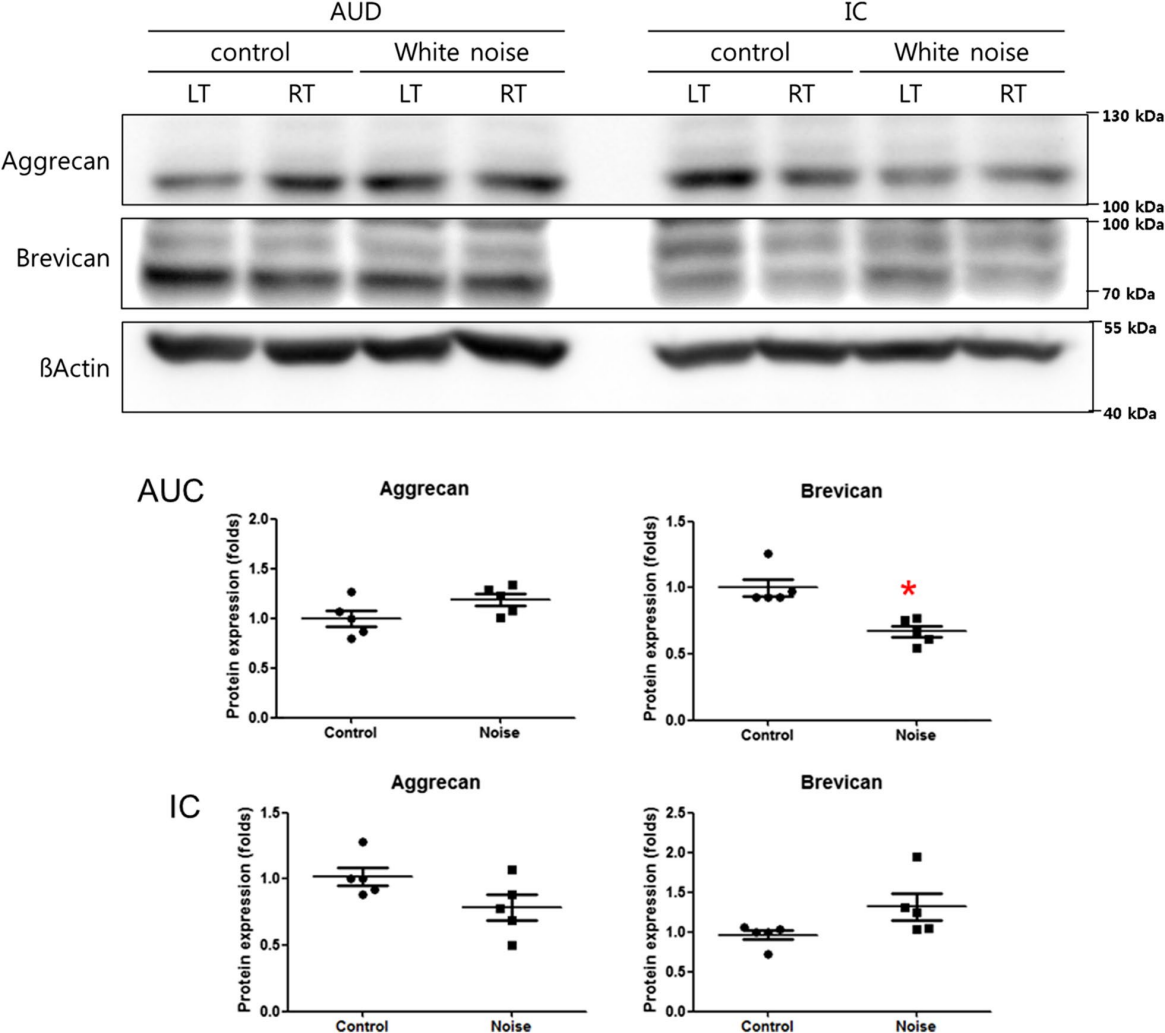
The brevican expression level in the primary auditory cortex was decreased in the noise group. The brevican expression level in the inferior colliculus was comparable between the noise and control groups. (*LT* left, *RT* right, *AUD* auditory cortex, *IC* inferior colliculus) (*P < 0.05)

**Fig. 5:**
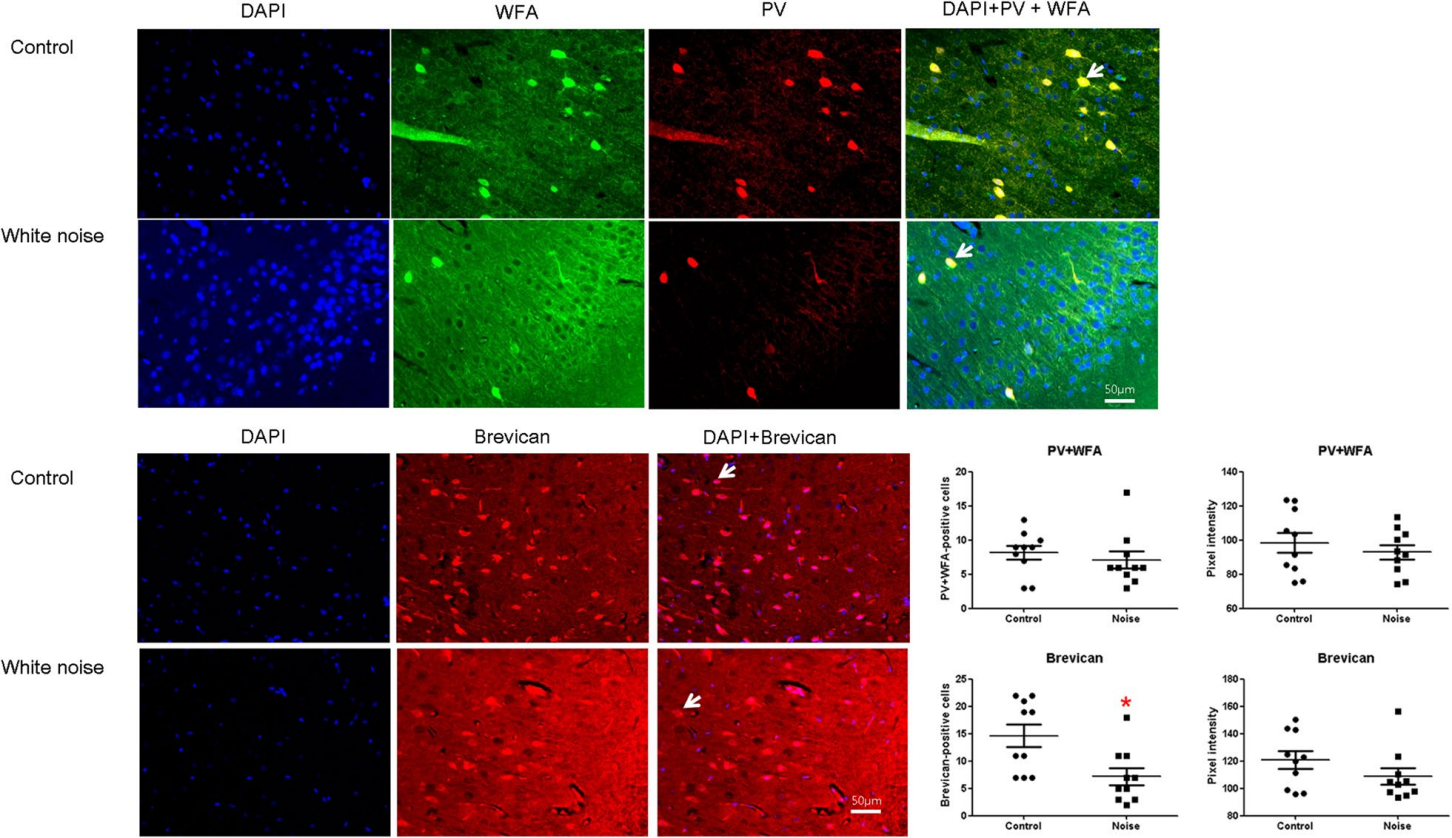
The perineuronal nets (PNNs) in the primary auditory cortex. The numbers of PV + WFA- and brevican-positive cells per 350 × 250 mm^2^ were decreased in the noise group compared with the control group. White arrow indicated co-localized cells. (*P < 0.05)

After noise exposure, the protein expression level of brevican was decreased in the primary auditory cortex (1.00 vs. 0.67 for the control vs. noise groups, P = 0.03 d.f. = 8, Mann–Whitney U test) (Fig. 4). However, in the inferior colliculus, brevican protein levels were not different between the noise and control groups. The expression level of aggrecan did not differ between the noise and control groups in either the primary auditory cortex or the inferior colliculus. The PV + WFA-positive cell density was lower in the noise group than in the control group (Fig. 5). Perinuclear brevican expression was also decreased in the noise group compared to the control group.

### Changes in the expression levels of matrix metalloproteinases

**Fig. 6:**
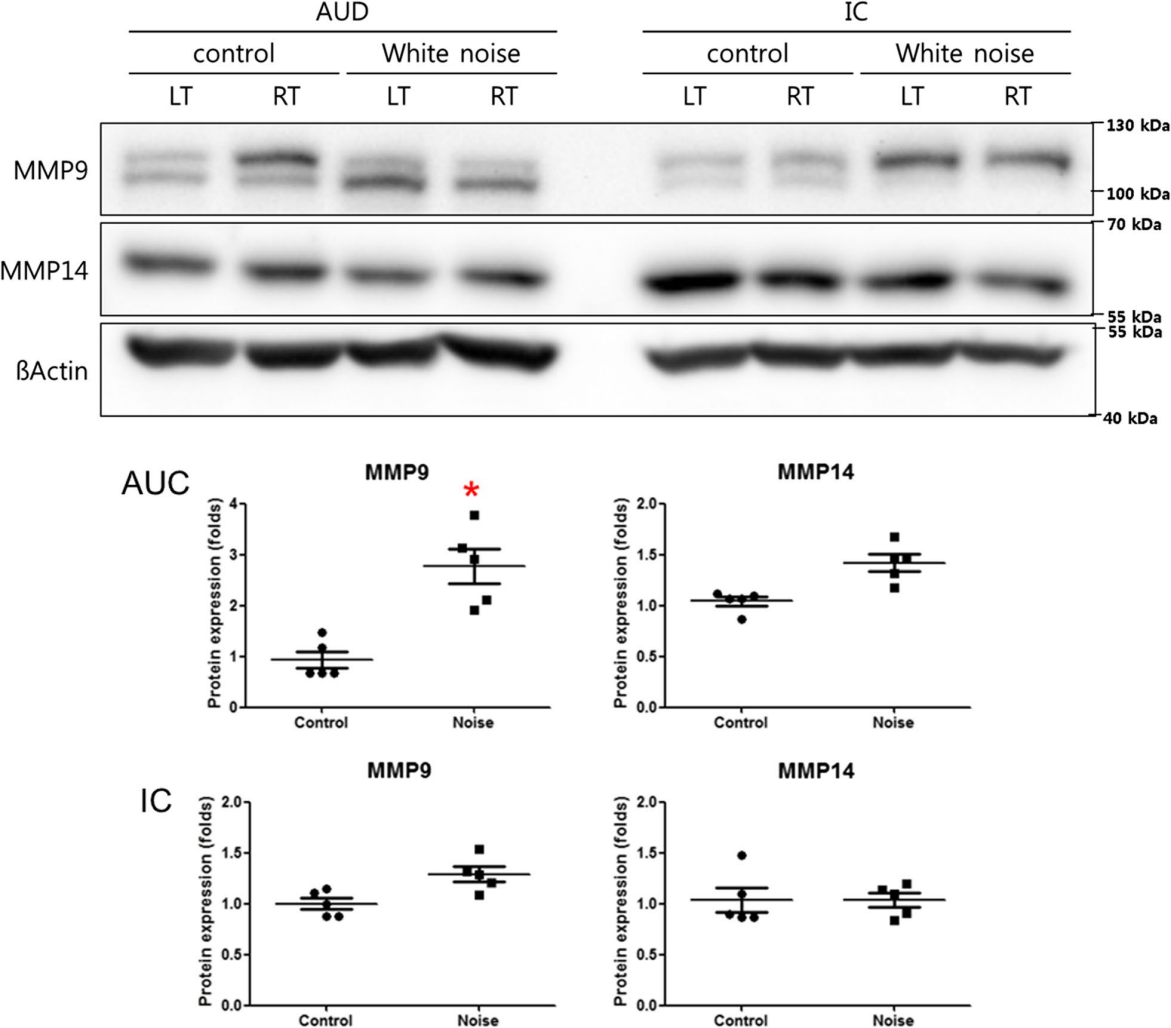
Matrix metalloproteinase (MMP)9 expression in the primary auditory cortex was increased in the noise group compared with the control group. MMP9 and MMP14 expression levels in the inferior colliculus were comparable between the noise and control groups. (*P < 0.05)

The protein expression level of MMP9 in the primary auditory cortex was increased in the noise group (0.99 vs. 2.99 for control vs. noise groups, P = 0.01 d.f. = 8, Mann–Whitney U test) (Fig. 6). The protein expression level of MMP14 in the primary auditory cortex was slightly increased in the noise group. The inferior colliculus showed comparable expression levels of MMP9 and MMP14 between the noise and control groups.

## Discussion

Glutamatergic neural transmission involving VGLUT1 and VGLUT2 was decreased 1 month after noise exposure in this study. GABAergic neural transmission did not show significant changes in the primary auditory cortex. The PNNs in the primary auditory cortex were reduced in the noise group compared with the control group. The expression level of brevican was decreased in the noise group. The elevated expression level of MMP9, which degrades the glycosaminoglycan side chains of chondroitin sulfate proteoglycans, may have contributed to the decrease in brevican and PNN expression. The inferior colliculus was demonstrated to have increased glutamatergic neural transmission of VGLUT2 in the noise group. However, the expression levels of the PNN components, such as brevican, and the degradative enzymes MMP9 and MMP4 did not change in the noise group. This finding might be attributed to the more rapid neural compensation to neuroplastic changes before 30 days after noise exposure in the subcortical area than in the cortical area. Moreover, the PNNs in the inferior colliculus were reported to be less relevant for VGAT expression, while PNNs were primarily related to VGAT in the primary auditory cortex [17]. Thus, the inhibitory synaptic plastic changes are not associated with the PNNs in the inferior colliculus.

The expression level of VGLUT1 in the primary auditory cortex was decreased in the noise group in the present study. During developmental periods, the expression level of VGLUT1 was reported to increase with age [18]. Similar to the present result, the expression level of VGLUT1 was decreased in the ventral cochlear nucleus of rats with age-related hearing loss [19]. The decreased VGLUT1 expression level in this study may imply reduced glutamatergic vesicular transmission after as long as 1 month after noise-induced hearing loss. On the other hand, the expression level of VGLUT2 was increased in the inferior colliculus after 1 month of noise-induced hearing loss in this study. Consistent with the present result, the expression level of VGLUT2 was elevated in the dorsal cochlear nucleus of subjects with unilateral cochlear deafness [20]. In the inferior colliculus, VGLUT2 is innervated with the nonauditory terminals of somatosensory and vestibular terminals, while the ipsilateral primary auditory cortex is the only source of VGLUT1 terminals [21]. Therefore, the increased expression of VGLUT2 in the inferior colliculus indicated the possibility of cross-modal compensation after hearing loss, which could be related to tinnitus [22].

The expression levels of both VGAT and GAD67 were similar in the noise and control groups in the present study. In the primary auditory cortex, the VGAT expression level was consistent after postnatal day 7 in a mouse study [18]. The GAD67 expression in noise-induced hearing loss has temporal differences depending on the duration of hearing loss [23, 24]. In the immediate early period, approximately 45 min after noise exposure, the GAD expression level was not changed in the primary auditory cortex [23]. Another animal study described decreased GAD67 expression at 4 days after noise-induced hearing loss, and this level recovered to the normal hearing control level at 32 days after noise-induced hearing loss [24]. Thus, the changes in GABAergic neurotransmission may have occurred before 30 days after noise exposure and then normalized at 30 days after noise exposure in this study. Although GABAergic neurotransmission in the noise group was comparable to that in the control group, PNN expression, which is mainly associated with inhibitory interneurons, was decreased in the primary auditory cortex in this study. The decrease in PNNs in the primary auditory cortex could have impacted the inhibitory synaptic function of animals in the noise group.

The expression level of brevican in the primary auditory cortex was decreased in the noise group in this study. Brevican is known to be related to neuroplasticity via multiple mechanisms [25]. Brevican is mostly localized in the proximity of the synapse in presynaptic, postsynaptic, and transsynaptic areas of the brain. In the synaptic area, brevican interacts with synaptic receptors, such as the GluR1 subunit of the AMPA receptor, to induce synaptic plasticity [26]. In addition, alternation of the sulfate state and the splicing of brevican were suggested to be related to neuroplastic changes [25]. In the auditory nervous system, brevican was reported to play a role in fast synaptic transmission, which was supported by a knockout mouse study [27]. In the calyx of Held, brevican knockout resulted in the delayed transmission of synaptic action potentials accompanied by a reduction in presynaptic VGLUT1 expression [28]. Thus, the decreased expression of brevican in the noise group might have influenced the downregulation of VGLUT1 expression in this study.

The expression level of MMP9 in the primary auditory cortex was increased in the noise group in this study. Consistent with the present result, the expression level of MMP9 in the primary auditory cortex was elevated in a model of age-related hearing loss [29]. The increased expression of MMP9 was also related to hearing loss in an acute brain injury model [30]. MMP9 regulated PNN expression by degrading chondroitin sulfate proteoglycans and permitted neuroplastic changes in the central nervous system in a knockout mouse study [31]. In addition, MMP9 indirectly regulates PNNs via the activation of advanced glycation end-products and inflammatory cytokine cascades [32]. The increased expression level of MMP9 in the noise group might have decreased PNN and brevican expression, thereby influencing the neuroplastic changes associated with auditory sensory deficits after noise exposure in this study. The modulation of these changes in PNNs could be a solution to alleviate hearing loss-related neuroplastic changes, such as tinnitus and hyperacusis. The underlying factors associated with these changes should be delineated in future studies. This study used female mice. Because it has been suggested that the auditory cortical responses during auditory stimulation including noise have sex-differences [33, 34], the possible differences in male are warrant to be considered.

## Conclusion

Noise exposure in the precritical period resulted in decreased PNNs accompanied by a decrease in the presynaptic excitatory transporter in the primary auditory cortex. The expression level of brevican was decreased in rats with noise-induced hearing loss, which was probably mediated by the increased expression level of MMP9 in the primary auditory cortex.

## Data Availability

The datasets generated and/or analyzed during the current study are available from the corresponding author upon reasonable request.

## Abbreviations

VGLUT1: Vesicular glutamatergic transporter
VGAT: Vesicular GABA transporter
GAD67: Glutamate decarboxylase
PV: Parvalbumin
WFA: *Wisteria floribunda* agglutinin
PNNs: Perineuronal nets
MMPs: Matrix metalloproteinases
ABRs: Auditory brainstem responses
TBS-T: Tris-buffered saline containing Tween-20
DAPI: 4′,6-diamidino-2-phenylindole
